# Induction of conidial traps in the nematode-trapping fungus *Drechslerella dactyloides* by soil microbes

**DOI:** 10.1128/msystems.01291-24

**Published:** 2025-02-13

**Authors:** Ling Zhang, Tao Zhang, Yan-Rui Xu, Jia-Mei Sun, Xue-Rong Pan, Kun-Ze Gu, Ke-Qin Zhang, Zhi-Gang Zhang, Lian-Ming Liang

**Affiliations:** 1 State Key Laboratory for Conservation and Utilization of Bio-Resources in Yunnan, Yunnan University 12635 , Kunming, China; 2 Translational Pharmaceutical Laboratory, Jining First People′s Hospital, Shandong First Medical University, Jining, China; University of California, Davis, Davis, California, USA

**Keywords:** nematode-trapping fungi, microbiome, conidial trap

## Abstract

Nematode-trapping fungi, renowned for their specialized predatory structures that ensnare nematodes, offer a promising biological approach to managing plant-parasitic nematodes. However, the efficacy of these fungi is frequently hampered by biotic and abiotic factors within the soil, which can significantly impede fungal germination (fungistasis). To counteract these environmental challenges, certain nematode-trapping fungi have evolved to produce traps from their conidia, referred to as conidial traps. This adaptation allows them to bypass the inhibitory effects of their surroundings, enhancing their predatory capabilities. In this study, we explored how soil affects conidial trap formation in *Drechslerella dactyloides*. Our findings revealed that *Acinetobacter* spp. and *Pantoea* spp. present in soil extracts play pivotal roles in triggering the development of these traps. Using metagenomic sequencing, we mapped the shifts in bacterial communities and their relative abundances before and after incubation for up to 24 hours to optimize soil induction effects. This analysis highlighted the enrichment of specific functional genes in soil microbes and provided insights into the mechanisms driving conidial trap formation, based on changes in soil characteristics. Furthermore, through bacterial isolation procedures, we successfully cultured and characterized the bacteria responsible for this phenomenon, confirming their potent ability to stimulate the production of conidial traps in nematode-trapping fungi. This study not only underscores the critical role of bacterial diversity in modulating the life cycle transitions of nematode-trapping fungi but also sets the stage for the development of more effective and sustainable strategies to harness these fungi in the battle against pathogenic nematodes.

**IMPORTANCE:**

Predatory nematode-trapping fungi are important microbial antagonists of nematodes and can be developed into biocontrol agents. However, microbial biocontrol agents often suffer from inconsistent efficacy, primarily due to biotic and abiotic stresses in the rhizosphere soil. *Drechslerella dactyloides*, a nematode-trapping fungus, produces conidial traps in soil, serving as a survival strategy to overcome these stresses. In this study, we optimized soil suspensions to efficiently induce the formation of conidial traps. We found that bacteria in the soil directly trigger this formation. Metagenomic sequencing revealed bacterial enrichment during optimization, and we isolated and purified these bacteria with inducible activity. Our research deepens the understanding of this survival strategy of nematode-trapping fungi in nature, laying the foundation for enhancing the effectiveness of nematode biocontrol using this mechanism.

## INTRODUCTION

Pathogenic fungi that infect humans, animals, and plants often have two distinct life stages: saprophytic and parasitic. Fungi show different growth phenotypes in these two life stages. For example, the human opportunistic fungal pathogen *Candida albicans* undergoes a yeast-to-hyphae transition (1). Entomopathogenic fungi from the genus *Metarhizium* can shift phenotypes from filamentous hyphae to unicellular blastospores (budding yeast-like structures) upon successful penetration into the hemocoel (body cavity) of an insect host (2, 3). A crucial aspect of a successful host invasion is the proper transition between these two life stages. This transition is dependent on the fungi’s perception of the host and its environment, followed by an appropriate response. The mechanisms underlying this response are currently a major focus of research on pathogenic fungal infections.

Nematode-trapping fungi are a group of fungi capable of producing specialized mycelial structures, known as traps, to effectively capture nematodes (4). They hold significant potential as bio-control agents against plant-parasitic nematodes, which cause substantial agricultural losses worldwide (5–7). The traps in different species of nematode-trapping fungi exhibit various types, including three-dimensional networks, adhesive knobs, adhesive branches, and constricting rings (8). The traps are the hallmark of the transition from the saprophytic to the parasitic life stage in these fungi (9, 10). Among these, the constricting rings are particularly notable for their precision, capable of rapidly contracting within a timeframe of 0.1–1 second (11).

The ability of nematode-trapping fungi to survive fungistatic, substances or conditions that inhibit fungal growth in the natural environment, is crucial to their successful establishment (12). Numerous unsuccessful colonization attempts occur due to this reason. Therefore, their adaptation capacity plays a vital role in their practical application for controlling plant-parasitic nematodes in the field (12). To enhance nematode-capturing capabilities and increase environmental adaptability, nematode-trapping fungi can directly generate traps from conidia, known as conidial traps (CTs) (13). The production of CTs is considered as an adaptive ability of nematode-trapping fungi to thrive in their environment (14).

Previous studies have indicated that water-saturated soil or soil extract can induce the formation of CTs in nematode-trapping fungi. Different fungi exhibit varying abilities to generate CTs, with the species *Drechslerella dactyloides*, which produces constricting-ring traps, surpassing all other fungi in CT production (13). However, the mechanism behind the induction of conidial trap formation by soil remains unclear. In this study, we first identified the crucial role of soil microorganisms in CT production. We optimized the conditions for inducing CT formation and then employed metagenomic approaches to investigate the taxonomy and associated biological functions involved in the induction of conidial traps. Through isolation and cultivation, we obtained several bacterial strains capable of inducing conidial trap production. Finally, we proposed potential mechanisms underlying the induction of conidial traps.

## RESULTS

### Bacteria in soil extract are key inducers of conidial trap formation

Previously, it was reported that soil extract can induce the formation of conidial traps in predatory nematode-fungal species, such as *D. dactyloides*, which produced thevmost conidial traps among different species (13). To verify the induction effect of soil extract, different soil samples were collected in the vicinity of Kunming City, China, including farmland, campuses, and other areas, for the induction of CT production in *D. dactyloides*. The results indicate that the inducibility of these soil samples is either low or unstable (data not shown). Therefore, it is crucial to explore a stable condition for inducing the production of conidial traps in *D. dactyloides*.

Subsequently, rhizosphere soil samples of nematode-infected tomato were employed to induce CTs. The induction of CTs was initially highly effective, but it remained unstable. In the subsequent experiments, we optimized the conditions for the soil extract. As illustrated in Fig. S1, three treatments were carried out for soil extracts. The sample filtered using a 0.45 µm filter and incubated for 24 hours had the highest efficiency for CT induction and the lowest germination rate. The soil extract that was neither filtered nor incubated showed moderate induction efficiency and germination rate, while the sample that was filtered but not subjected to incubation had the lowest effect and the highest germination rate. Furthermore, this condition’s stability was confirmed in subsequent experiments.

**Fig 1:**
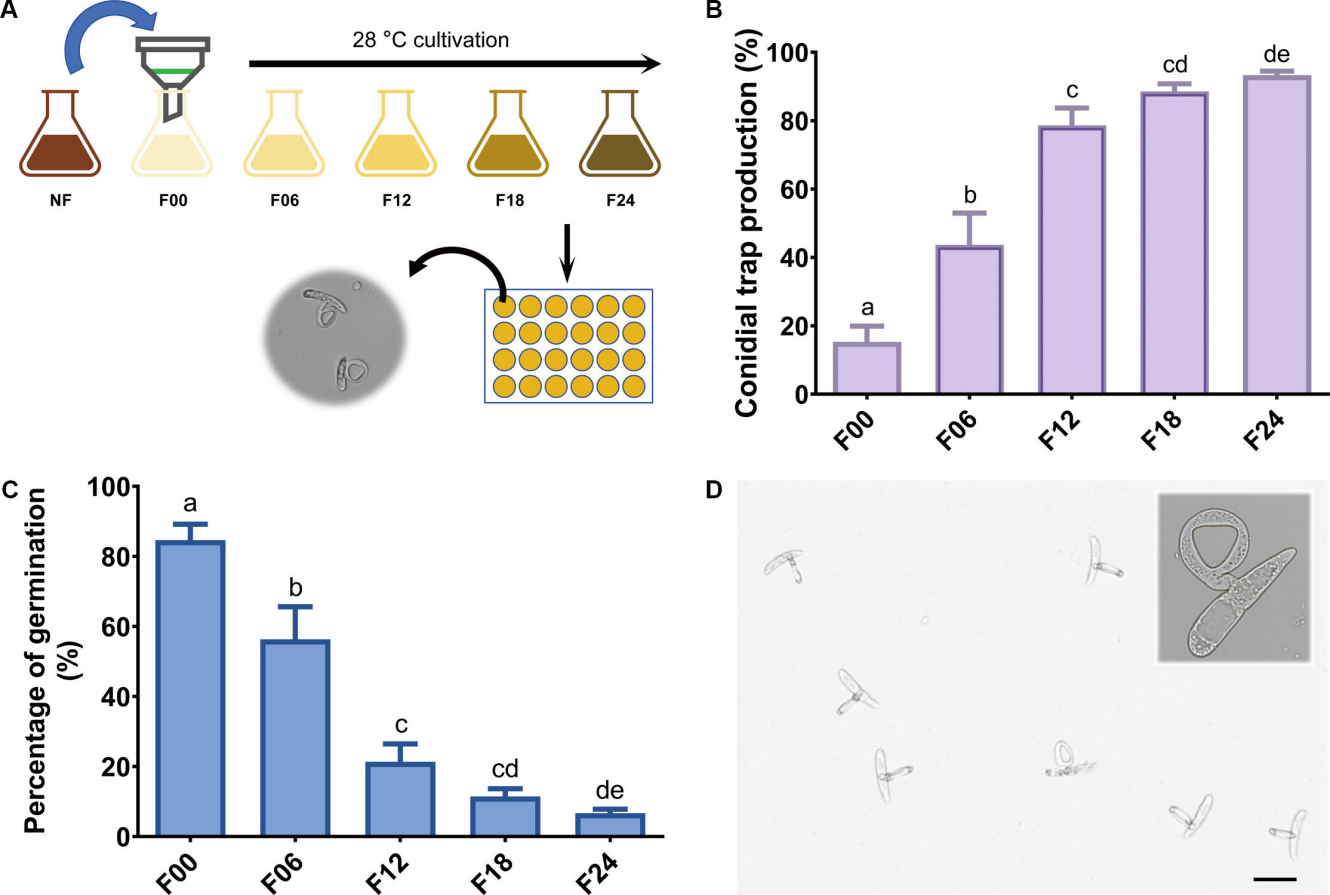
Soil extract-induced conidial trap formation. (**A**) Optimization of the process for soil extract-induced conidial trap development. The soil extract was first filtered through a 0.45 µm pore size membrane filter, followed by incubation at 28°C for 24 hours, with samples taken every 6 hours to induce conidial trap and calculate induction rates. NF, soil extracts without filtration and incubation; F00-F24, soil extracts incubated for 0, 6, 12, 18, and 24 hours. (**B**) Yield of conidial trap induced by different soil extract samples. (**C**) Conidia germination rates induced by soil extract with varying durations of incubation. In panels B and C, different lowercase letters above columns indicate statistical differences as *P* < 0.05 according to ordinary one-way ANOVA test. (**D**) Conidial trap induced by a 24-hour-incubated soil extract. Bar = 50 µm.

To confirm that incubation contributed to a higher induction effect of soil extracts, pre-incubation on a shaker was performed for 0, 6, 12, 18, and 24 hours (Fig. 1A). Soil extracts from each time point were collected, and the induction effect was examined. With increasing pre-incubation time, the induction efficiency of the soil extract improved. After 12 hours of incubation, the induction rate reached approximately 80%, while the 18-hour incubation yielded a rate of around 90%. The most effective induction was observed after a 24-hour pre-incubation, resulting in an induction rate exceeding 93% (Fig. 1B). As the production rate of CTs increased, the germination rate (formation of normal mycelium) gradually declined (Fig. 1C). Meanwhile, the filtrate obtained by filtering each time point’s soil extract through a 0.22 µm filter was also measured. The filtrate obtained from the 0-hour pre-incubation and filtered through a 0.22 µm filter did not show any induction effect on CTs in *D. dactyloides*. With an extended pre-incubation time, the filtrate gradually acquired low-level induction capability (Fig. S2A), although most of the conidia were germinated (Fig. S2B). This indicates that bacteria play a major role in the induction of CTs in the soil extract.

### Specific bacteria accumulated during the optimization of soil extracts

To study the bacteria and their functions in soil extracts, samples were taken from both filtered and unfiltered soil extracts, and total DNA was extracted for metagenomic sequencing. Each sample had five replicates, resulting in a total of 30 samples (Fig. 1A). After performing quality control on the raw data and removing host contamination, a total of 257 GB of clean data were obtained, with an average of 8.6 GB of valid data per sample.

**Fig 2:**
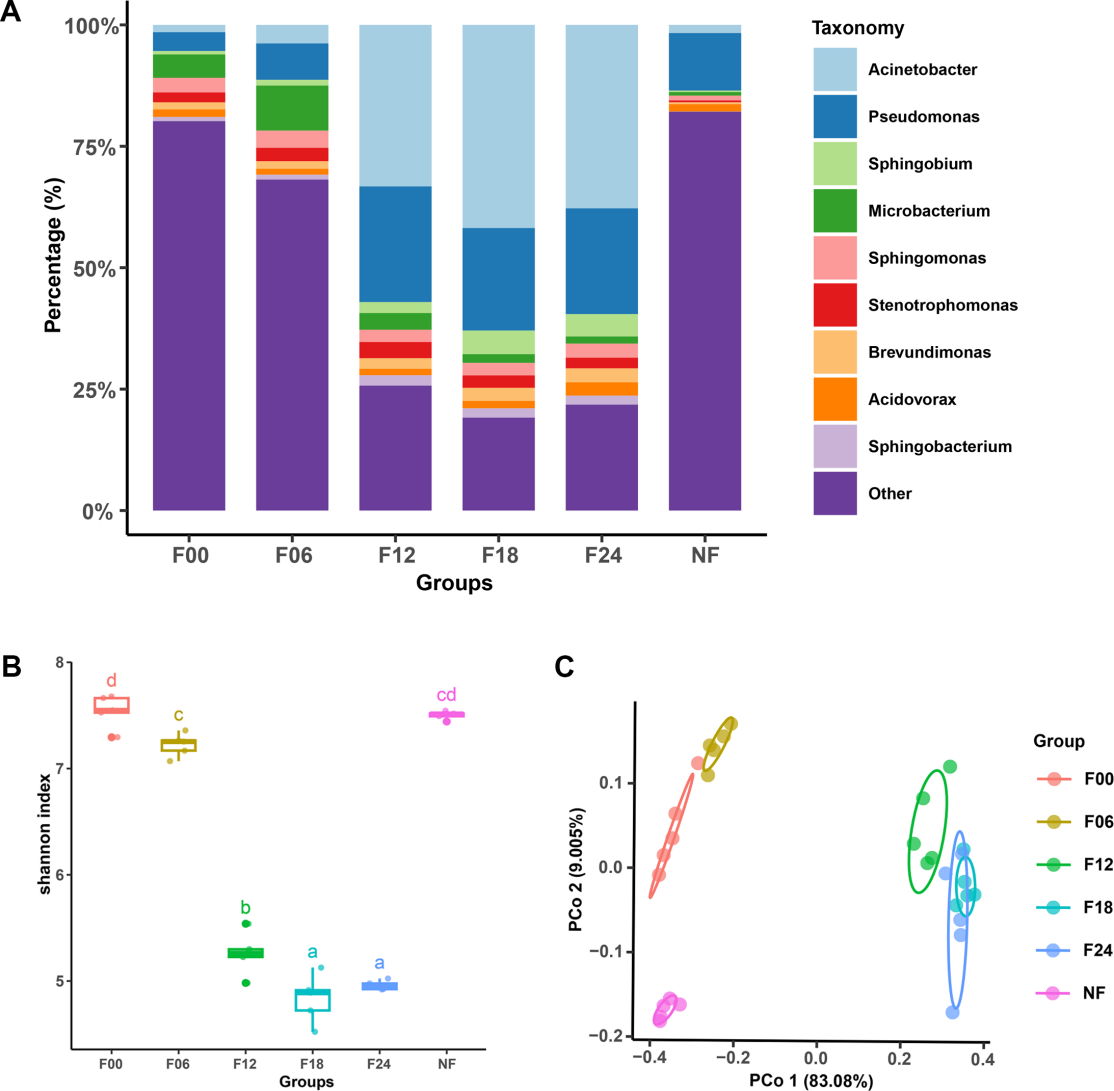
The biodiversity in soil extracts that induce conidial traps in *D. dactyloides*. (**A**) The main genera in the soil extracts. (**B**) Alpha diversity comparison between soil extracts. (**C**) Beta diversity of bacteria in soil extracts analyzed by PCoA method.

**Fig 3:**
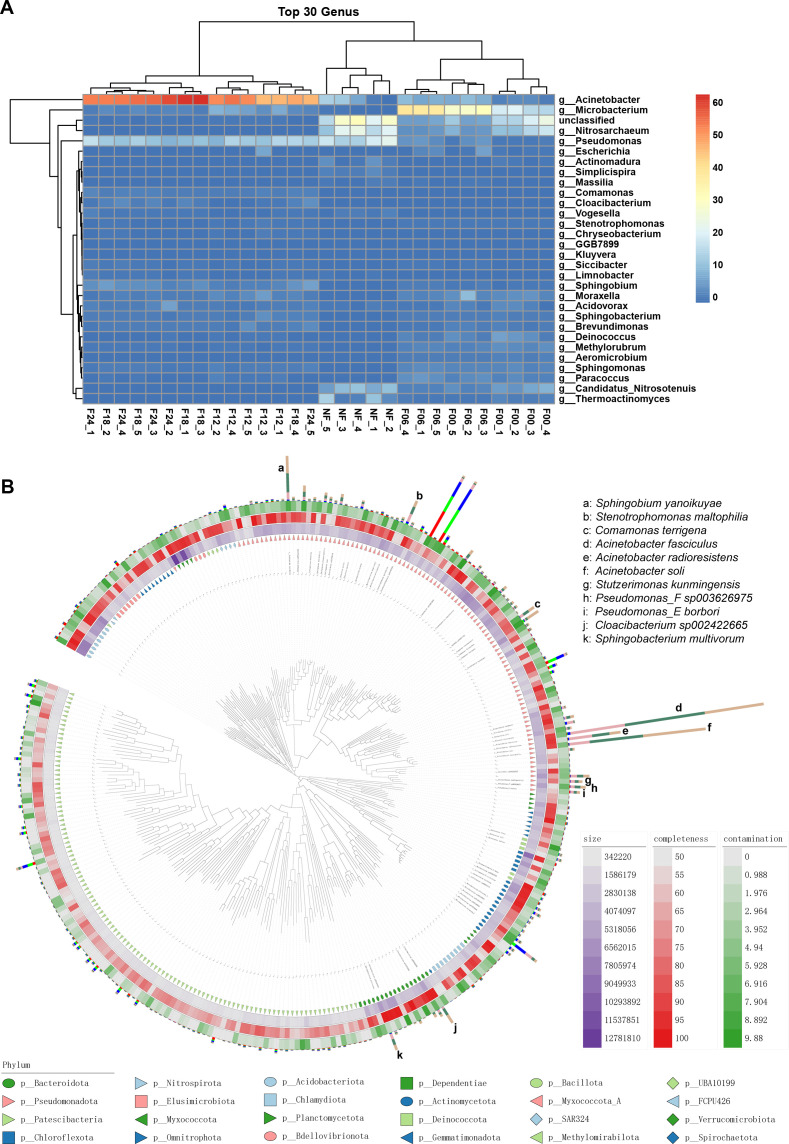
Enrichment of bacteria in soil extract samples. (**A**) Heatmap of top 30 genera in the samples. (**B**) The phylogenetic tree of binning metagenomes. Out of the tree are the phyla, the genome sizes, the completeness, and contamination.

The composition of the bacteria species was significantly different between each sample group (Fig. 2A). Most species of the samples belonged to the genera *Acinetobacter*, *Pseudomonas*, *Sphingobium*, *Microbacterium*, *Sphingomonas*, and *Stenotrophomonas*, etc. Specifically, bacteria belonging to the genera *Acinetobacter* (*P* = 0.000095, KW *H* test), *Pseudomonas* (*P* = 0.000137, KW *H* test), and *Sphingobium* (*P* = 0.000046, KW *H* test) exhibited significant enrichment and dominance in the samples after being incubated for 12–24 hours (Fig. 2A and 3A). The alpha diversity analysis showed a dramatic decrease in Shannon and other indexes during incubation, indicating the incubation reduced species richness and evenness of the soil extract (Fig. 2B). For the beta diversity, the principal coordinate analysis (PCoA) based on Bray-Curtis distance showed that each sample in each treatment was grouped together. We can observe that PCo1 coordinates account for 83% of the variation, indicating a clear separation between the soil extract samples incubated for 12–24 hours and the other three sample groups (Fig. 2C). The soil extract samples incubated for 18 and 24 hours are essentially clustered together, indicating a close diversity of prokaryotes between them, which is consistent with their similar conidial trap-inducing ability.

Binning was conducted using MaxBin and MetaBAT2, resulting in 983 and 678 bins, respectively. These bins were then subjected to dereplication at the species level using dRep, with criteria set at 99% similarity, completeness greater than 50%, and contamination less than 10%. Ultimately, 337 species-level genome bins (SGBs) were obtained. These metagenome-assembled genomes (MAGs) were further classified and annotated using GTDB-tk based on the presence of 120 universal single-copy proteins (bac120) commonly found in bacteria. Of these, 51 SGBs could be confidently assigned to species, while the remaining 85% of SGBs were classified only to higher taxonomic ranks, indicating the presence of a substantial number of unidentified and relatively abundant bacteria (compared to those not captured by MAGs) in the soil samples. Quantification of bins was performed using CoverM, revealing enrichment of certain bacteria, such as those belonging to the genera *Acinetobacter*, *Pseudomonas*, and *Sphingobacterium* in samples incubated for 12–24 hours (Fig. 3B).

### Metagenomic analysis reveals key pathways associated with conidial trap formation in soil extracts

**Fig 4:**
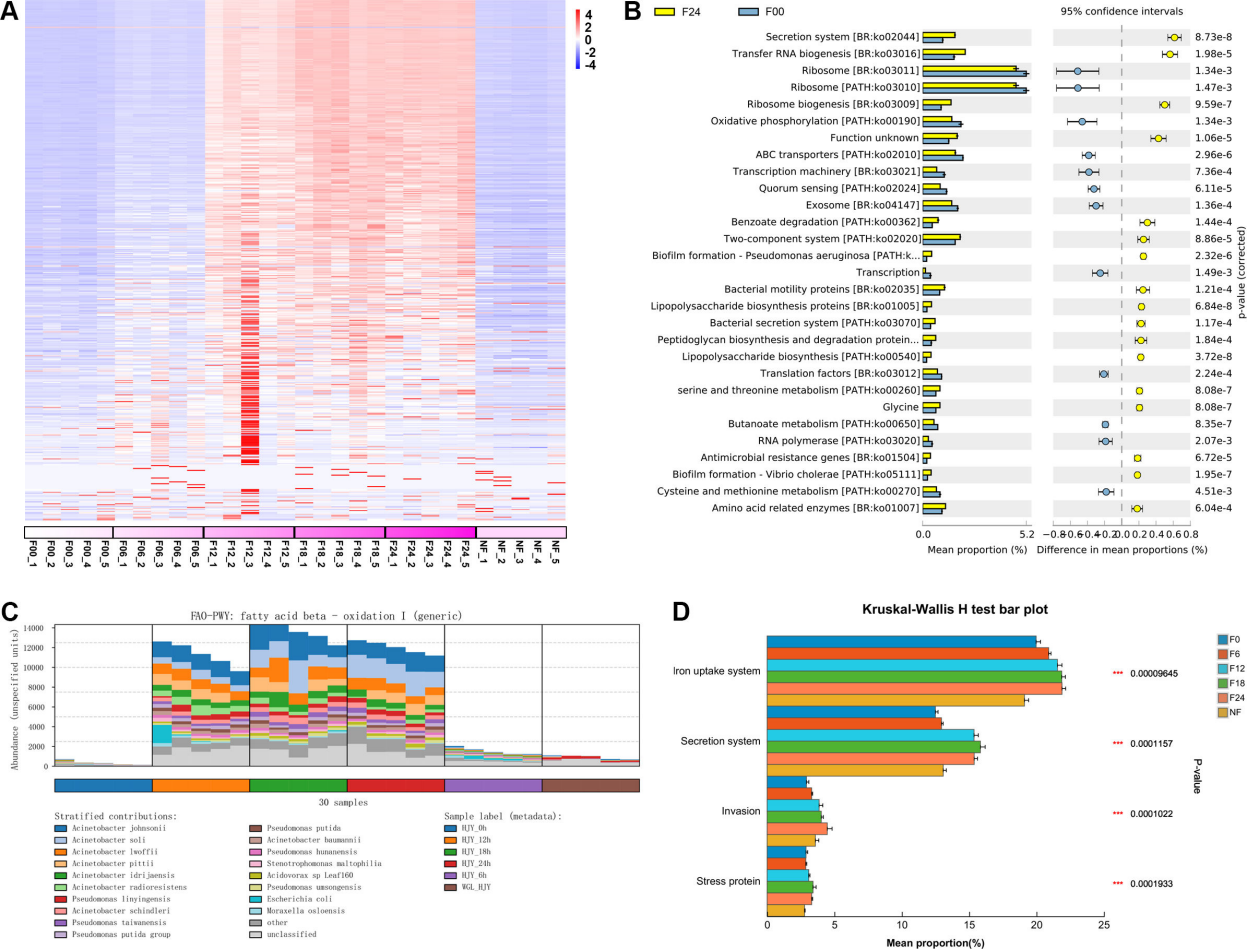
Functional comparison between different soil extract samples. (**A**) Heatmap of KEGG pathways among different samples. Each column represents one sample, and each row represents one pathway; colors represent pathway abundances. The purple bands represent relative activities of each group of soil extracts to induce ring formation by *D. dactyloides*. (**B**) Pathway abundance comparison by F00 and F24 samples. (**C**) The fatty acid beta-oxidation pathway was contributed by different bacterial species. (**D**) The most different virulence factors between the soil extract samples.

Using the Humann3 software, reads were aligned against the database, resulting in the generation of a species composition table and a pathway composition table. The Stamp software was used to perform a comparative analysis of pathway abundances. After sorting based on eta-squared, a heatmap of pathway abundances was plotted (Fig. 4A). The results revealed significant differences in KEGG pathways among the samples incubated for 12, 18, and 24 hours, and the unfiltered and filtered samples incubated for 0 and 6 hours. The pathways that were particularly enriched in the samples incubated for 12, 18, and 24 hours included biofilm formation, transfer RNA biogenesis, secretion system, lipopolysaccharide biosynthesis, antimicrobial resistance genes, lipid metabolism, and thyroid hormone synthesis. We selected the 0- and 24-hour-incubated samples for comparison and found that the pathways significantly enriched at 24 hours included secretion system, transfer RNA biogenesis, benzoate degradation, two-component system, biofilm formation, and lipopolysaccharide biosynthesis. Conversely, pathways such as ribosome, ABC transporters, transcription machinery, transcription, and RNA polymerase showed significant decreases (Fig. 4B). This indicates that the enriched species obtained through incubation and cultivation are associated with species adaptation and inter-species interactions.

Using Metacyc enrichment analysis, it was also discovered that the fatty acid metabolism pathways and the linoleic acid and arachidonic acid metabolic pathway were enriched in the 12–24 hour-incubated samples. The enriched pathways were mainly contributed by bacteria, such as *Acinetobacter johnsonii*, *Acinetobacter soli*, *Acinetobacter lwoffii*, *Pseudomonas linyingensis*, *Pseudomonas taiwanensis*, *Pseudomonas putida*, and *Escherichia coli* (Fig. 4C). The enrichment of fatty acid metabolism pathways suggests the presence of active fatty acid metabolism processes in the samples. The accumulation of linoleic acid and arachidonic acid indicates relatively higher levels of these specific fatty acids in the samples. Linoleic acid and arachidonic acid are polyunsaturated fatty acids that play essential roles in the structure and function of cell membranes. Furthermore, these fatty acids have been reported to possess fungicidal properties (15), and their ability to induce conidial trap formation in D. *dactyloides* requires further investigation.

By querying the VFDB database for virulence factors and performing a Kruskal-Wallis *H* test for inter-group comparison, it was observed that with the prolonged incubation time of soil extracts after filtration, certain bacterial virulence factor pathways became increasingly enriched. These pathways include the iron uptake system, secretion system, invasion, and stress protein, among others (Fig. 4D; Fig. S3). These pathways are potentially critical for the interaction between bacteria and filamentous *D. dactyloides*, inhibiting germination and inducing the production of conidial traps.

### Nutrients deprived during soil extract optimization

**Fig 5:**
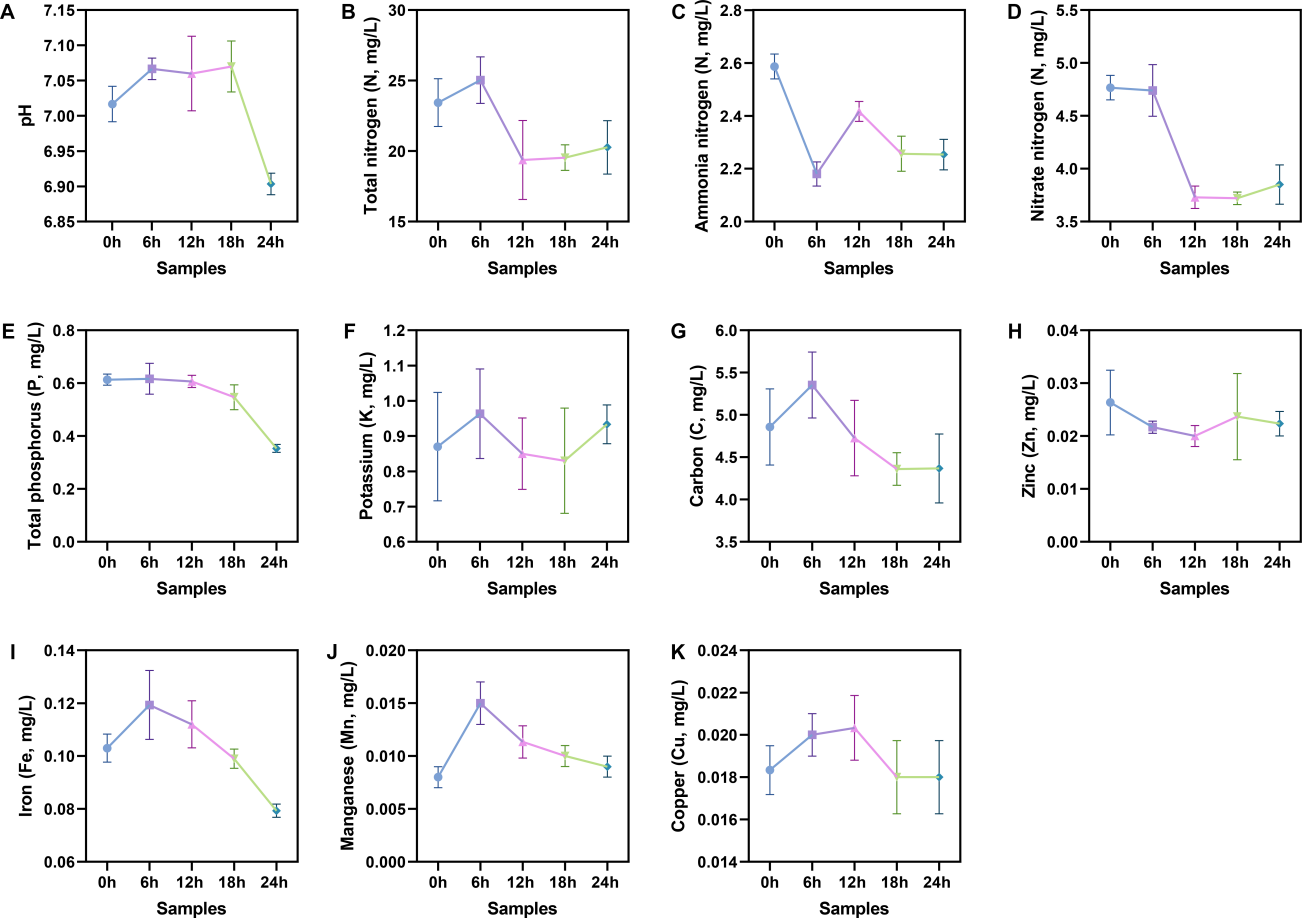
Physicochemical profiles of different soil extract samples. (A) pH; (B) total nitrogen; (C) ammonia nitrogen; (D) nitrate nitrogen; (E) total phosphorus; (F) potassium; (G) carbon; (H) zinc; (I) iron; (J) manganese; (K) copper.

The above analysis revealed that one of the reasons why bacteria can induce the formation of CTs in *D. dactyloides* may be that bacteria can compete with *D. dactyloides* for nutrients, leading to a nutrient-limited induction system. The lifestyle of *D. dactyloides* will shift from saprophytic to predacious, thereby inducing the formation of conidial traps. Therefore, 11 soil parameters of the soil extracts were measured. From Fig. 5, it can be observed that total nitrogen, total phosphorus, nitrate nitrogen, iron, carbon, and other parameters showed a decreasing trend with the prolongation of pre-cultivation time. Among them, the iron content decreased by 33.3%, total nitrogen decreased by 20%, nitrate nitrogen decreased by 16%, and carbon decreased by nearly 20%. It can be concluded that the production of CTs in *D. dactyloides* was related to the nutrient limitation in the soil extract.

### Accumulated bacteria were linked to the induction of conidial traps

We considered whether a single bacterium could induce the formation of conidial traps in *D. dactyloides*. To do this, we isolated and cultivated 220 bacterial strains from the incubated soil extracts, as described in Materials and Methods. The species were identified based on their 16S rDNA sequences. Individual bacterial strains re-suspended in 0.22 µm microporous membrane-filtered soil extract were used to induce CT formation in *D. dactyloides*.

**Fig 6:**
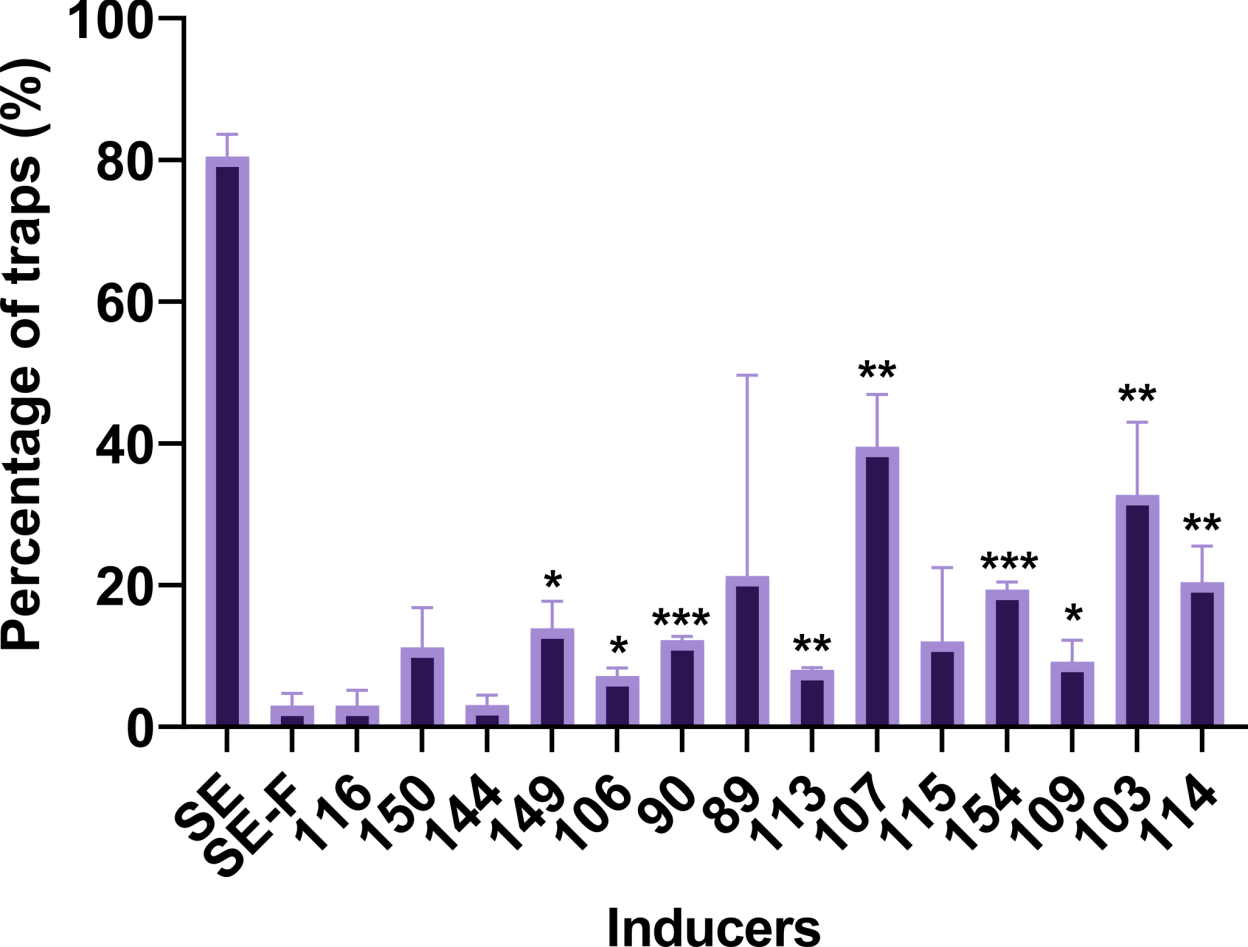
Induction of conidial traps by soil extract and various bacterial strains. SE, soil extract; SE-F, filtered soil extract; numbers in *X* axis represent strain numbers. *P < 0.05; **P < 0.01; and ***P < 0.001. *t*-test for inducing rate of each inducer compared to SE-F.

The specific activities of the bacteria are shown in Fig. 6. Among the tested bacteria, strains 89, 90, 114, 149, and 154 showed an induction rate of around 20% for the conidial trap, while strains 107 and 103 exhibited an induction rate of approximately 40%. The conidia that did not form CTs almost all germinated. Among them, strains 89, 114, and 154 belonged to the genus *Acinetobacter*, while strains 103 and 107 belonged to the genus *Pantoea* and were identified as the main contributors to the induction of conidial trap.

## DISCUSSION

It is a common phenomenon among filamentous fungi that certain specialized hyphal structure can directly differentiate from spores without going through a nutritional mycelium stage. In addition to nematode-trapping fungi, which can form traps from their conidia, other examples include plant and animal parasitic fungi. For instance, the conidia of the rice blast fungus (*Magnaporthe oryzae*) can produce appressorium when they attach to leaf surfaces (16). Furthermore, fungal species subjected to stress conditions exhibit an extremely simplified asexual life cycle, in which the conidia that germinate directly generate further conidia, without forming mycelia. This phenomenon has been termed microcycle conidiation, and to date, it has been reported in more than 100 fungal species (17). These structures indicate the strong differentiating ability of the conidia themselves. At the same time, this differentiation appears to be a product of conidia perception, adaptation to the environment, and interaction with the environment, reflecting the rapid adaptability of fungi (the destiny of differentiation may be determined before germination).

The conidia of the nematode-trapping fungus *D. dactyloides* can directly germinate under certain conditions to produce conidial traps. In this study, a stable method for inducing the formation of CTs in *D. dactyloides* was established. Bacterial diversity analysis was conducted on pre-incubated soil extract samples at different time intervals, such as 0, 6, 12, 18, and 24 hours. Metagenomic analysis revealed that the bacterial community changed as pre-incubating time increased. *Acinetobacter*, *Pseudomonas*, *Sphingomonas*, and some other genera increased in abundance after 24 hours of pre-incubation, becoming the dominant groups. Unclassified members of Beta, Gamma, Alpha Proteobacteria, and Anaerolineales gradually decreased in abundance with prolonged pre-incubation time, and the unclassified genus *Anaerolineales* disappeared during the pre-incubation process. The induction capacity of the soil extracts reached its peak after 24 hours of pre-incubation, indicating the need to consider the bacterial composition after 24 hours of pre-culturing. The majority of the identified active strains were *Acinetobacter* spp. and *Pantoea* spp., which were the main contributors to the induction of CT formation.

The main functional categories identified through KEGG annotation at each pre-culturing stage include amino acid metabolism, carbohydrate metabolism, coenzyme and vitamin metabolism, energy metabolism, membrane transport, signal transduction, microbial community—prokaryotes, nucleotide metabolism, translation and synthesis/metabolism of polysaccharides, biosynthesis of secondary metabolites, biosynthesis of coenzymes, citric acid metabolism, and amino sugar and nucleotide sugar metabolism. Among these, *Acinetobacter* and *Pseudomonas* were found to contribute the most to the above-mentioned functions after 24 hours of pre-culturing.

We compared the physicochemical parameters of the clarified supernatant obtained by filtering the soil extracts during the pre-culturing process. We found that as the pre-culturing time increased, there was a decrease in pH. Additionally, the levels of total nitrogen, ammonium nitrogen, nitrate nitrogen, and total phosphorus all decreased, indicating a gradual reduction in nutrients in the soil extracts, consistent with the enriched metabolic pathways identified in the metagenomic analysis. The iron content also gradually decreased, corresponding to the observed enhancement of the iron uptake system in the metagenomic analysis. These findings suggest that the deprivation of nutrients such as nitrogen, phosphorus, and iron may be potential factors inducing CT formation in the nematode-trapping fungus.

The nematode-trapping fungi are highly susceptible to environmental stress in agricultural soil, which hinders its effective germination and limits its application as a biocontrol agent against nematodes (12). To address this issue, it is crucial to understand the interactions between the nematode-trapping fungus, other organisms, and abiotic factors in the rhizosphere soil environment. Currently, there is a lack of systematic research in this area. Building upon previous studies that induced conidial trap formation in the nematode-trapping fungus through soil and soil extract (13), this study further clarifies the significant role of bacteria in soil as the main inducer. Bacteria play a critical role in nutrient cycling and decomposition in natural ecosystems, and some bacteria face predation pressure from bacterivorous nematodes. In response to this predation pressure, bacteria have evolved various defense mechanisms (18). For example, bacteria like *Stenotrophomonas maltophilia* can interact with the nematode-trapping fungus *Arthrobotrys oligospora* by producing and releasing urea, thereby promoting the formation of traps to reduce predation pressure (19). In this study, we found that the main CT induction effect was attributed to the direct interaction between bacteria and fungi, while the bacteria-free soil extract only slightly induced CT formation. From the metagenomic study, we have revealed the enriched bacteria of soil extracts during incubation and isolated several bacteria that can induce the formation of CTs in *D. dactyloides*. The identification of these bacteria lays the foundation for further elucidating the interactions between bacteria and nematode-trapping fungi and may facilitate the utilization of these fungi to control plant and animal parasitic nematodes. In the future, we will focus on studying the molecular mechanisms by which individual bacteria induce the formation of conidial traps in *D. dactyloides*.

### Conclusion

In this study, we uncover the bacterial-fungi interaction that facilitates lifestyle transition of the nematode-trapping fungus *D. dactyloides*. This group of fungi evolved the strategy of conidial trap formation under stressful circumstances. Further research into the mechanism underlying bacterially induced conidial trap formation would be beneficial to harness the power of nematode-trapping fungi to manage plant-parasitic nematodes.

## MATERIALS AND METHODS

### Preparation of soil extract

A total of 1,000 g of rhizosphere soil from tomato plants infected with root-knot nematodes (*Meloidogyne incognita*) for 2 months was mixed with deionized water at a 1:1 (wt/vol) ratio. The mixture was thoroughly mixed and settled for 24 hours. The supernatant was collected by centrifuging at 1,000 rcf for 5 minutes and filtering using a 0.45 µm microporous membrane. A volume of 100 mL of the filtrate was transferred to a 250 mL Erlenmeyer flask and shaken under 180 rpm at 22°C for 24 hours or other time intervals. Three replicates were performed for each preparation.

### Preparation of conidia of *D. dactyloides*

Mycelial chunks grown on potato dextrose agar (PDA) plates for approximately 7 days were inoculated into flasks containing cornmeal agar solid media. After incubating at 28°C for 12–15 days, conidia were harvested using sterile water and sterile glass beads and filtered through six layers of 10 × 15 cm lens-cleaning paper (Newstar, Hangzhou, China) to obtain fresh conidia.

### Induction of conidial traps

In a 48-well plate, 1 × 10^6^ fresh conidia were mixed with 200 µL of soil extract. Subsequently, the 48-well plate was placed in a constant temperature incubator at 22°C and allowed to incubate undisturbed for 24 hours. Conidial trap formation and conidia germination were examined and counted under a light microscope. Each treatment was performed in triplicate.

### Physicochemical property testing of soil extracts

The soil extract samples were sent to the Yunnan Academy of Agricultural Sciences for testing according to the requirements. The measured parameters include pH, total nitrogen, total phosphorus, potassium, ammonia nitrogen, nitrate nitrogen, copper, zinc, iron, manganese, and carbon of the soil extracts. Pre-cultured samples were subjected to sterilization using 0.22 µm filter paper at five different time intervals: 0, 6, 12, 18, and 24 hours. Each sample was tested in triplicate.

### Metagenomic sequencing and analysis

Soil extract samples were filtered through a microporous membrane to collect all the microbes present in them. Then, the microporous membranes were ground in liquid nitrogen, and total DNA was isolated using the FastDNA Spin Kit for Soil (MP Biomedicals). Concentration and purity of extracted DNA were determined by TBS-380 and NanoDrop2000, respectively. DNA extract quality was checked on 1% agarose gel. A paired-end library was constructed using NEXTFLEX Rapid DNA-Seq (Bioo Scientific, Austin, TX, USA). Then, the sequencing was performed on an Illumina Novaseq platform. The raw reads were quality controlled by FastQC (version 0.12.0) and Trimmomatic (version 0.40) (20). Then, the clean reads were analyzed with the EasyMetagenome Pipeline (version 1.20) (21, 22). Briefly, the read-based analysis was first carried out by HUMAnN (version 3.0) and MetaPhlAn (version 4.0.6) to get taxonomy and gene abundance. Kraken (version 2.1.3) and Bracken (version 2.6.2) were used for taxonomic annotation and abundance estimation (23, 24).

For the binning, the clean reads from all 30 samples were first assembled into contigs using MEGAHIT (version 1.2.9) (25). The mixed sample binning process was performed using Metawrap (version 1.3) (26), utilizing Metabat2 and Maxbin2 algorithms along with the clean reads from all 30 samples and their assembled contigs (27, 28). Bin refinement was conducted to select bins with completeness greater than 50% and contamination below 10%. Quantification of bins across different samples was accomplished using the “metawrap quant_bins” command. Bin dereplication was carried out using the dRep program (version 3.4.2), employing a 99% strain-level dereplication threshold (29). Species annotation and phylogenetic tree construction of the bins were performed using GTDB-Tk (version 2.3.2) (30).

### Isolation and culturing of bacteria from soil extract and CT induction

The 24-hourincubated soil extract was diluted 50 times, and 50 µL was spread on different bacterial isolation media as previously reported (31). The plates were incubated at 28°C for 20 hours. After incubation, individual colonies were transferred to slants. Meanwhile, 16S rDNA universal primers 27F: AGAGTTTGATCCTGGCTCAG and 1492R: TACGGCTACCTTGTTACGACTT were used to perform 16S rDNA amplification and sequencing for each bacterial isolate (32). The sequencing results were analyzed on https://www.ezbiocloud.net/identify to identify the corresponding bacterial species.

The identified bacteria were inoculated into LB medium and incubated on a shaker at 28°C and 180 rpm for 15 hours. The bacteria were then washed with a 0.22 µm filtered soil extract (without incubation) to remove LB medium and diluted to the same concentration as the soil extract. A 10 µL aliquot containing 1 × 10^6^ conidia/mL of *D. dactyloides* was mixed with 200 µL of the bacterial suspension and co-cultured statically at 22°C for 24 hours. After 24 hours, 10 µL of the co-culture was taken for microscopic examination. Each experiment was performed in triplicate.

### Statistical analyses

The differences in the conidial trap production rate and conidial germination rate under different soil extract treatments were examined using an ordinary one-way ANOVA test using the GraphPad Prism software (version 9.5.0). A comparison of conidial traps induced by soil extract and various bacterial strains was carried out by *t*-test using the GraphPad Prism software (version 9.5.0). Virulence factor genes were mapped against the Virulence Factors of Pathogenic Bacteria database (33), and their gene abundance between the soil extract samples was analyzed for differences using the Kruskal-Wallis *H* test, with computations performed on the Majorbio Cloud Platform (https://cloud.majorbio.com/).

## Data Availability

Raw sequencing data generated in this project are available in the Sequence Read Archive (SRA) with the BioProject accession number PRJNA1165823.

## SUPPLEMENTAL MATERIAL

Supplemental figures msystems.01291-24-s0001.docx Fig. S1-S3.

ASM does not own the copyrights to Supplemental Material that may be linked to, or accessed through, an article. The authors have granted ASM a non-exclusive, world-wide license to publish the Supplemental Material files. Please contact the corresponding author directly for reuse.
