# Supplementary material for: Induction of conidial traps in the nematode-trapping fungus Drechslerella dactyloides by soil microbes
Source: mSystems. 2025 Feb 13;10(3):e01291-24. doi: 10.1128/msystems.01291-24 (PMC11915823; doi:10.1128/msystems.01291-24)
Supplement: Supplemental figures — Fig. S1-S3. [file msystems.01291-24-s0001.docx]

**Supplementary materials**

# Induction of conidial traps in the nematode-trapping fungus *Drechslerella dactyloides* by soil microbes

Ling Zhang^1†^, Tao Zhang^1,2†^, Yan-Rui Xu^1^, Jia-Mei Sun^1^, Xue-Rong Pan^1^, Kun-Ze Gu^1^, Ke-Qin Zhang^1^, Zhi-Gang Zhang^1^*, Lian-Ming Liang^1^*

^1^ State Key Laboratory for Conservation and Utilization of Bio-Resources in Yunnan, Yunnan University, Kunming, China.

^2^ Translational Pharmaceutical Laboratory, Jining First People’s Hospital, Shandong First Medical University, Jining, China.

^†^ Ling Zhang and Tao Zhang contributed equally to this work.

*Correspondence: [lianglm@ynu.edu.cn](mailto:lianglm@ynu.edu.cn); zhangzhigang@ynu.edu.cn


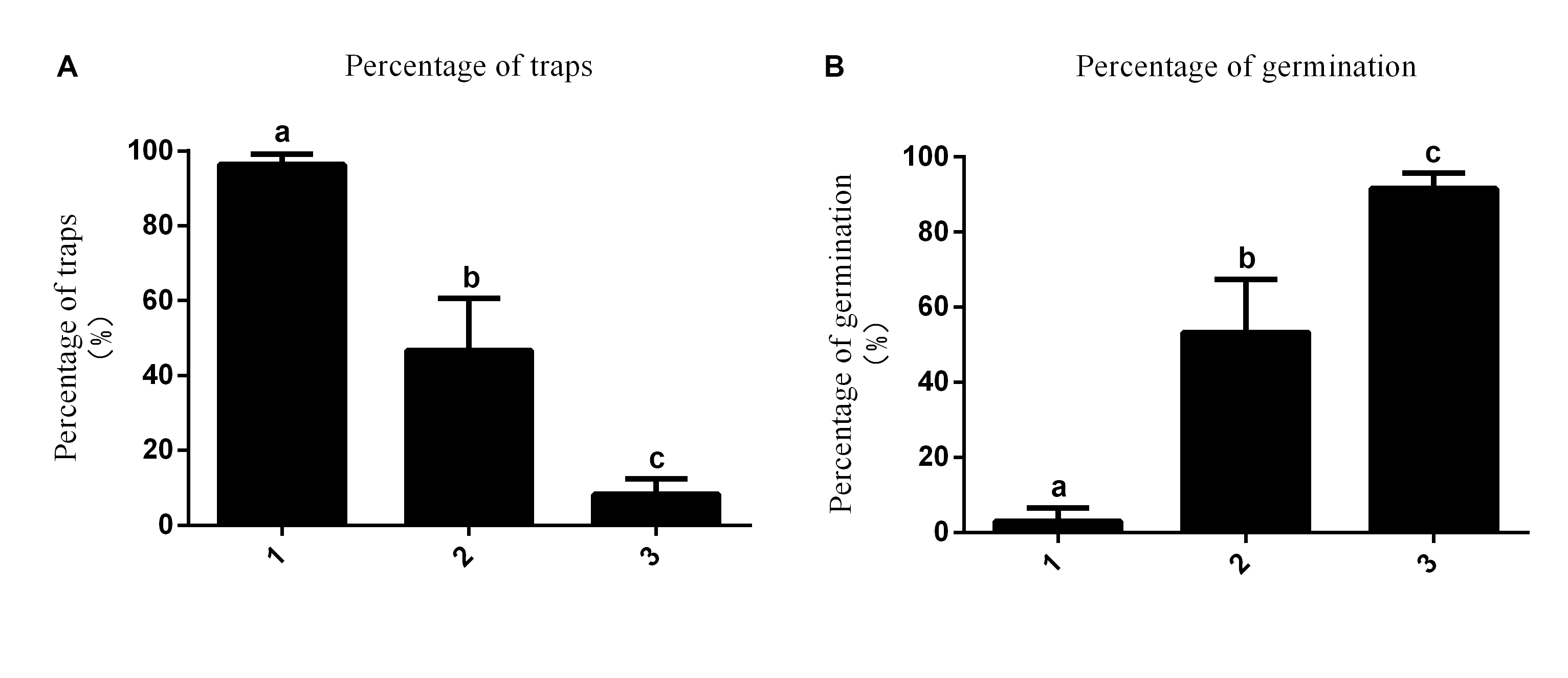


Fig.S1 Optimization of soil extracts for inducing conidial trap production. (A) Induction rate of conidial trap induced by different soil extract samples. (B) Conidia germination rates induced by different soil extract samples. The samples were treated as described in the main text. 1, soil extract was filtered using a 0.45 μm filter and then subjected to shaking at 22°C and 180 rpm for 24 hours prior to conidial trap induction. 2, soil extract was neither filtered nor shaken. 3, soil extract was filtered through a 0.45 μm filter but not subjected to shaking. Different lowercase letters above columns indicate statistical differences as p < 0.05 according to ordinary one-way ANOVA test.


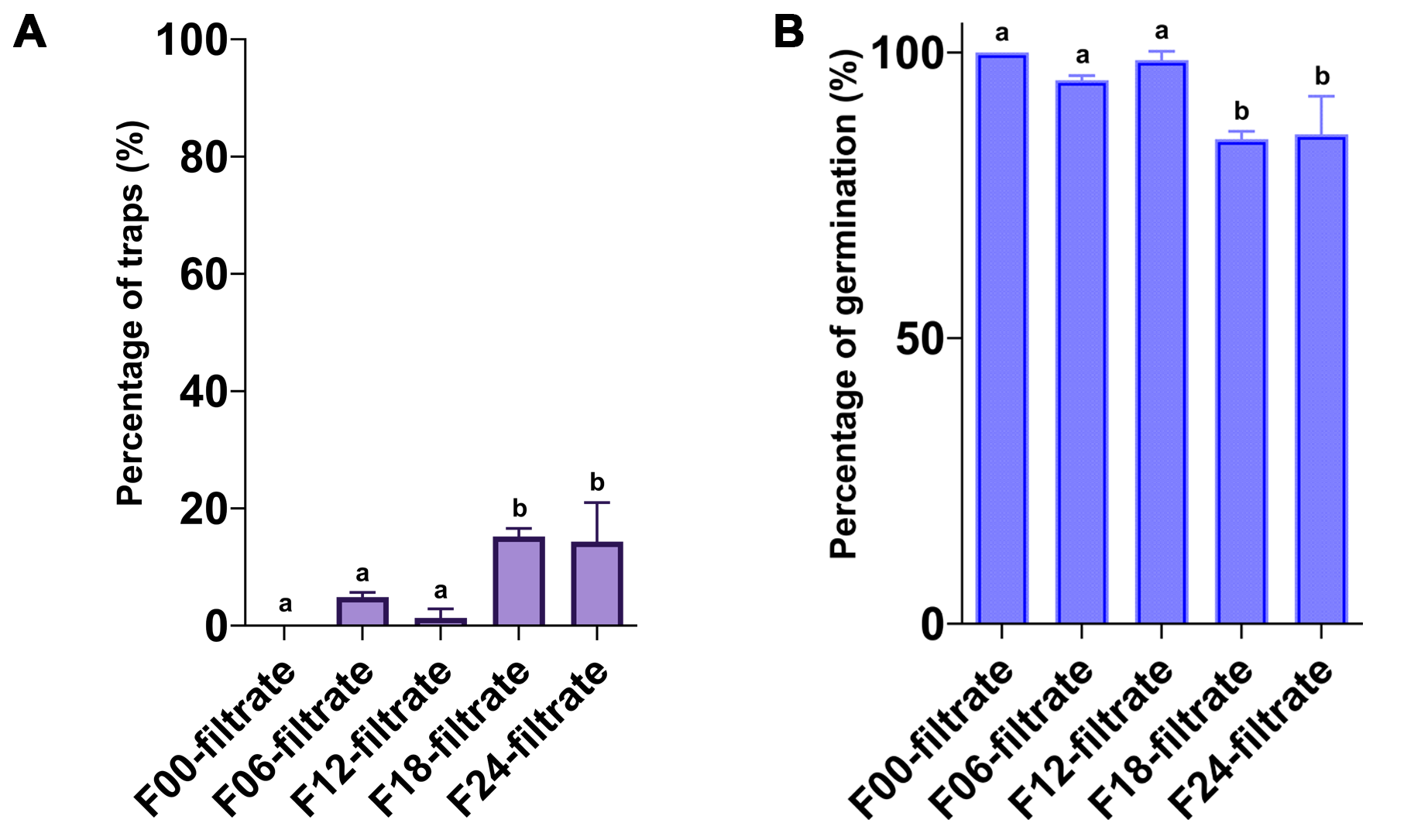


Fig. S2 Induction of CTs (A) and germination of conidia (B) induced by soil extract without bacterial. The supernatants of soil extracts were obtained by filtering samples F00 to F24, and the induction of conidial traps and conidial germination were examined. Different lowercase letters above columns indicate statistical differences as p < 0.05 according to ordinary one-way ANOVA test.


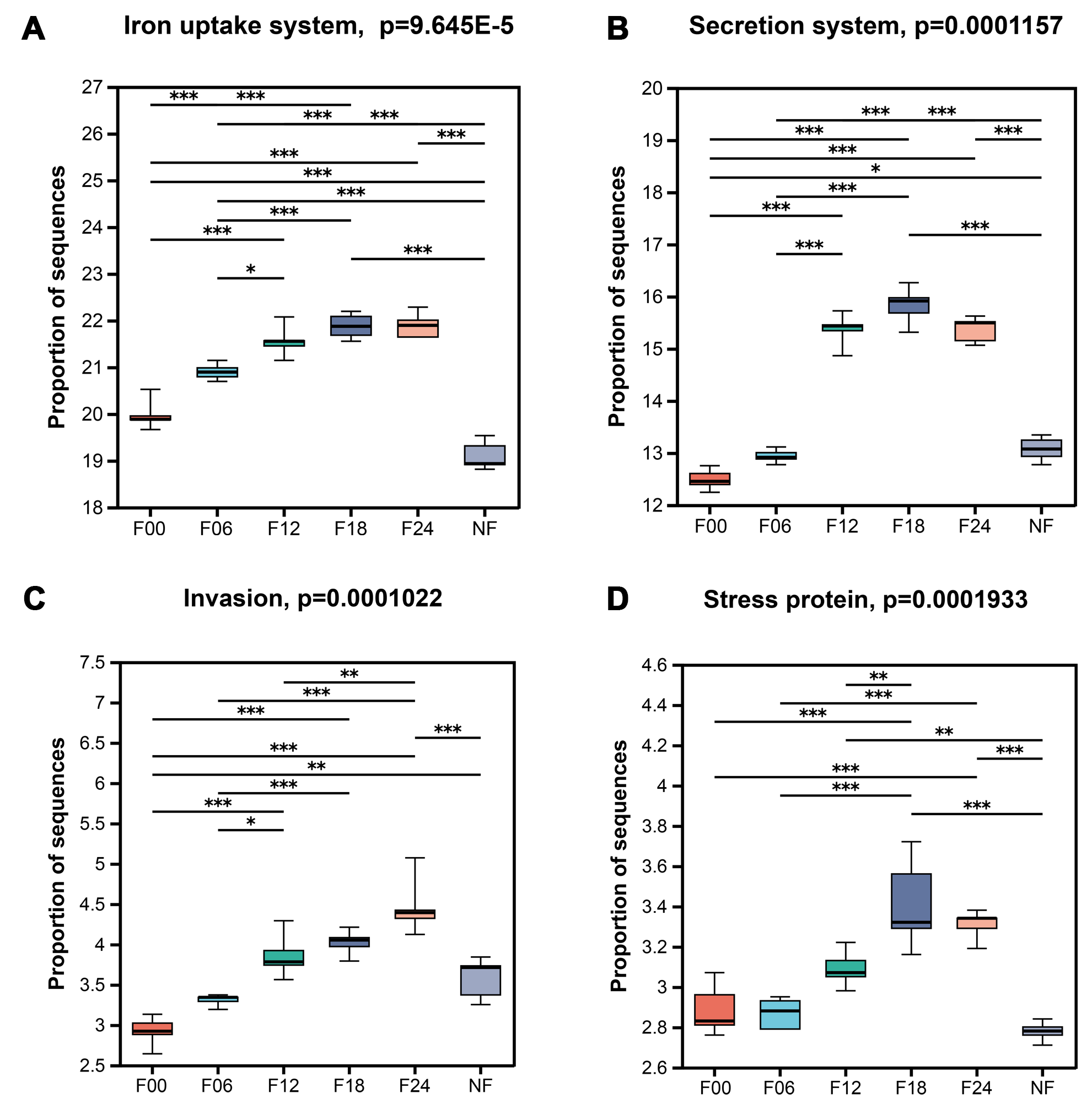


Fig. S3 virulence factors enrichment analysis for microbiota in different soil extracts.
